# Supplementary material for: Waist circumference and mortality or cardiovascular events in a general Korean population
Source: PLoS One. 2022 Apr 27;17(4):e0267597. doi: 10.1371/journal.pone.0267597 (PMC9045645; doi:10.1371/journal.pone.0267597)
Supplement: Attachment — Submitted filename: Response_to_Reviewers.docx [file pone.0267597.s001.docx]

PONE-D-21-39364

Waist circumference and mortality or cardiovascular events in a general Korean population

PLOS ONE

Dear Dr. Lee,

Thank you for submitting your manuscript to PLOS ONE. After careful consideration, we feel that it has merit but does not fully meet PLOS ONE’s publication criteria as it currently stands. Therefore, we invite you to submit a revised version of the manuscript that addresses the points raised during the review process.

The revised version should address all comments.

Please submit your revised manuscript by Mar 25 2022 11:59PM. If you will need more time than this to complete your revisions, please reply to this message or contact the journal office at [plosone@plos.org](mailto:plosone@plos.org). When you're ready to submit your revision, log on to https://www.editorialmanager.com/pone/ and select the 'Submissions Needing Revision' folder to locate your manuscript file.

Please include the following items when submitting your revised manuscript:

- A rebuttal letter that responds to each point raised by the academic editor and reviewer(s). You should upload this letter as a separate file labeled 'Response to Reviewers'.
- A marked-up copy of your manuscript that highlights changes made to the original version. You should upload this as a separate file labeled 'Revised Manuscript with Track Changes'.
- An unmarked version of your revised paper without tracked changes. You should upload this as a separate file labeled 'Manuscript'.

If you would like to make changes to your financial disclosure, please include your updated statement in your cover letter. Guidelines for resubmitting your figure files are available below the reviewer comments at the end of this letter.
→ Thank you for your kind advice. We inserted the statement of our financial disclosure as “The authors received no specific funding for this work.” in our cover letter.

If applicable, we recommend that you deposit your laboratory protocols in protocols.io to enhance the reproducibility of your results. Protocols.io assigns your protocol its own identifier (DOI) so that it can be cited independently in the future. For instructions see: <https://journals.plos.org/plosone/s/submission-guidelines#loc-laboratory-protocols>. Additionally, PLOS ONE offers an option for publishing peer-reviewed Lab Protocol articles, which describe protocols hosted on protocols.io. Read more information on sharing protocols at <https://plos.org/protocols?utm_medium=editorial-email&utm_source=authorletters&utm_campaign=protocols>.

We look forward to receiving your revised manuscript.

Kind regards,

Petri Böckerman
Academic Editor
PLOS ONE

Journal Requirements:
1. When submitting your revision, we need you to address these additional requirements.

Please ensure that your manuscript meets PLOS ONE's style requirements, including those for file naming. The PLOS ONE style templates can be found at
https://journals.plos.org/plosone/s/file?id=wjVg/PLOSOne_formatting_sample_main_body.pdf and
https://journals.plos.org/plosone/s/file?id=ba62/PLOSOne_formatting_sample_title_authors_affiliations.pdf
[Note: HTML markup is below. Please do not edit.]
→ Thank you for your kind advice. As you recommended, we ensure that our manuscript meets PLOS ONE's style requirements, including those for file naming.

Reviewers' comments:

Reviewer's Responses to Questions
**Comments to the Author**

1. Is the manuscript technically sound, and do the data support the conclusions?

The manuscript must describe a technically sound piece of scientific research with data that supports the conclusions. Experiments must have been conducted rigorously, with appropriate controls, replication, and sample sizes. The conclusions must be drawn appropriately based on the data presented.
Reviewer #1: Yes
Reviewer #2: Yes

2. Has the statistical analysis been performed appropriately and rigorously?
Reviewer #1: Yes
Reviewer #2: Yes

3. Have the authors made all data underlying the findings in their manuscript fully available?

The [PLOS Data policy](http://www.plosone.org/static/policies.action#sharing) requires authors to make all data underlying the findings described in their manuscript fully available without restriction, with rare exception (please refer to the Data Availability Statement in the manuscript PDF file). The data should be provided as part of the manuscript or its supporting information, or deposited to a public repository. For example, in addition to summary statistics, the data points behind means, medians and variance measures should be available. If there are restrictions on publicly sharing data—e.g. participant privacy or use of data from a third party—those must be specified.
Reviewer #1: Yes
Reviewer #2: Yes

4. Is the manuscript presented in an intelligible fashion and written in standard English?

PLOS ONE does not copyedit accepted manuscripts, so the language in submitted articles must be clear, correct, and unambiguous. Any typographical or grammatical errors should be corrected at revision, so please note any specific errors here.
Reviewer #1: Yes
Reviewer #2: Yes

5. Review Comments to the Author

Please use the space provided to explain your answers to the questions above. You may also include additional comments for the author, including concerns about dual publication, research ethics, or publication ethics. (Please upload your review as an attachment if it exceeds 20,000 characters)
Reviewer #1: In this paper, the authors examined the associations between waist circumference (WC) and mortality or CVD incidence in a subset of Korean National Health Insurance Service cohort. They found that CVD incidence, but not mortality, was positively associated with WC in both men and women.
The paper is interesting for the ample number of subjects studied; nevertheless, it arises several observations that the authors should address:

1) It seems that the groups are not well balanced in participant numerosity, being the lowest WC group 38% of the total sample, whereas the highest WC group represents only 6% of it.
→ Thank you for your kind comment. In this study, quintiles for WC were not obtained. Furthermore, men and women were not distinguished in the analysis of basic characteristics. “Although the threshold for risky WC is suggested for men and women separately, the reference WC was set to less than 80 cm for both men and women in our study. Additional analysis setting different reference values of WC for men and women separately is required.” had been inserted in the eighth paragraph entitled “Discussion”.

2) In the majority of the international consensus (e.g.: IDF’s), the threshold for risky WC for Asian population is set in 80 cm for women and 90 cm for men. Therefore:
- a) In men, choosing the group with range 85-90 cm would have been more appropriate as a reference, rather than <80 category. Indeed, a low (< 80 cm) WC in men could be due to underlying disease, malnutrition or different socioeconomic aspects that could affect subject’s health.

→ Thank you for your kind comment. As you recommended, we fully agree that additional analysis is required by setting different reference values of WC for men and women separately. We excluded subjects who had a past history of cancers, stroke, ischemic heart, or chronic obstructive pulmonary disease or who died within one year from the start of the study because underlying diseases may affect the mortality results. Therefore, we think subjects with underlying disease or malnutrition are unlikely to have been included in our study. However, it is possible that different socioeconomic aspects could affect the results of our study. “Because we did not adjust the socioeconomic state that could affect subject’s health, it is possible that the different socioeconomic aspects had acted as confounding variables in our study.” has been inserted in the eighth paragraph entitled “Discussion”.

- b) The J or U shaped curve is a common finding in the relationships in which weight or other anthropometric variables related to nutritional status are considered. The point is to look at the nadir of the curve and consider if it falls within a “physiologic” status or not.

→ Thank you for your kind comment. To date, there have been several studies about the association between WC and mortality; however, the results have been inconsistent as described in the discussion. The results of our study had shown that WC exhibited a significant reverse J-shaped association with overall mortality in men. We excluded subjects who had a past history of cancers, stroke, ischemic heart, or chronic obstructive pulmonary disease or who died within one year from the start of the study because underlying diseases may affect the mortality results. We think that excluding higher risk subjects from the analysis makes the nadir of the curve about the association between WC and mortality or cardiovascular events to the “physiologic” status in our study. However, additional confounding factors such as the different socioeconomic aspects or other serious diseases could potentially affect the associations between WC and mortality.

- c) The lack of correlation between overall mortality and WC in women could be due to the fact that WC measurement does not distinguish between visceral fat and subcutaneous fat deposition in the belly (the last one being a more benign type of fat).

→ We appreciate for your advice. As you recommended, “The lack of correlation between overall mortality and WC in women could be due to the fact that WC measurement does not distinguish between visceral fat and subcutaneous fat deposition in the belly. Visceral fat accumulation is widely regarded as a risk factor for cardiovascular diseases and subcutaneous fat is regarded a more benign type of fat.” had been inserted sixth paragraph entitled “Discussion”.

3) Perhaps the decision of excluding people who died within the first year could have reduced higher risk subjects from the analysis.

→ Thank you for your kind comment. “Excluding subjects who died within the first year could have reduced higher risk subjects from the analysis.” has been inserted in the seventh paragraph entitled “Discussion”.

4) It is not known the WC at the moment of the event (death or ACV accident): this data would had given firmer conclusions.

→ Thank you for your kind comment. We used the cohort database released by the NHIS including individuals older than 40 years who were invited to participate in a biannual health checkup. “Because the NHIS cohort data were not collected for our study, it was impossible to know the WC at the moment of the event.” has been inserted in the eighth paragraph entitled “Discussion”. “Further research setting different reference values of WC for men and women separately and including changes in WC over the follow-up period is needed.” has been inserted in the last paragraph entitled “Discussion”.

5) Figures:
In fig 1, the horizontal axis is very expanded and the results are presented as cubic spline interpolation, whereas in fig. 2 the data are presented as points. Please explain the reason of such a different presentation.

→ Thank you for your kind comment. Figure 1 represents the mortality according to WC as a continuous variable, while Figure 2 shows the CVD incidence according to WC as a categorical variable. We had changed the “Association between waist circumference categories and overall mortality and CVD mortality as a cubic spline after adjusting for age” to “Association between waist circumference as a continuous variable and overall mortality and CVD mortality as a cubic spline after adjusting for age” in the title of figure 1.

Reviewer #2: It is an interesting article, but the following points need to be corrected.
1. The authors reported that they have received funding for this study. However, the source of fund was not mentioned in the article

→ Thank you for your kind comment. We changed the answer of the question about Financial Disclosure from “The funders had no role in study design, data collection and analysis, decision to publish, or preparation of the manuscript.” to “The authors received no specific funding for this work.”

2. Abstract: is preferred to write the full word of acronym when it first appears in the text.

→ Thank you for your kind comment. As you recommended, we changed WC to waist circumference (WC) when it first appears in the abstract.

3. Introduction: ok
4. Methods: ok
5. Results: make the tables and figures self-explanatory by including all necessary thing in the title

→ Thank you for your kind comment.

We had changed “Demographic and clinical characteristics of the subjects” to “Demographic and clinical characteristics of the subjects according to waist circumference categories” in the title of table 1.

Figure 1 represents the mortality according to WC as a continuous variable, while Figure 2 shows the CVD incidence according to WC as a categorical variable. We had changed the “Association between waist circumference categories and overall mortality and CVD mortality as a cubic spline after adjusting for age” to “Association between waist circumference as a continuous variable and overall mortality and CVD mortality as a cubic spline after adjusting for age” in the title of figure 1.

6. Discussion: OK
7. Conclusion: the conclusion is in line with the result, but what would the author recommended for the future researchers?

→ Thank you for your kind advice. As you recommended, “Further research setting different reference values of WC for men and women separately and including changes in WC over the follow-up period is needed.” has been inserted in the last paragraph entitled “Discussion”.

6. PLOS authors have the option to publish the peer review history of their article ([what does this mean?](https://journals.plos.org/plosone/s/editorial-and-peer-review-process#loc-peer-review-history)). If published, this will include your full peer review and any attached files.

If you choose “no”, your identity will remain anonymous but your review may still be made public.

**Do you want your identity to be public for this peer review?** For information about this choice, including consent withdrawal, please see our [Privacy Policy](https://www.plos.org/privacy-policy).
Reviewer #1: **Yes:** Raffaele Carraro Casieri
Reviewer #2: No
 

[NOTE: If reviewer comments were submitted as an attachment file, they will be attached to this email and accessible via the submission site. Please log into your account, locate the manuscript record, and check for the action link "View Attachments". If this link does not appear, there are no attachment files.]

While revising your submission, please upload your figure files to the Preflight Analysis and Conversion Engine (PACE) digital diagnostic tool, <https://pacev2.apexcovantage.com/>. PACE helps ensure that figures meet PLOS requirements. To use PACE, you must first register as a user. Registration is free. Then, login and navigate to the UPLOAD tab, where you will find detailed instructions on how to use the tool. If you encounter any issues or have any questions when using PACE, please email PLOS at [figures@plos.org](mailto:figures@plos.org). Please note that Supporting Information files do not need this step.

→ Thank you for your kind comment. As you recommended, we uploaded our figure files to the Preflight Analysis and Conversion Engine (PACE) digital diagnostic tool.
